# The efficacy of foot orthoses in individuals with patellofemoral osteoarthritis: a randomised feasibility trial

**DOI:** 10.1186/s40814-019-0469-7

**Published:** 2019-07-11

**Authors:** Jade M. Tan, Hylton B. Menz, Kay M. Crossley, Shannon E. Munteanu, Harvi F. Hart, Kane J. Middleton, Anne J. Smith, Natalie J. Collins

**Affiliations:** 1 Discipline of Podiatry, School of Allied Health, Human Services and Sport, La Trobe University, Melbourne, 3086 Australia; 2 La Trobe Sport and Exercise Medicine Research Centre, School of Allied Health, Human Services and Sport, La Trobe University, Melbourne, 3086 Australia; 3 Discipline of Sport and Exercise Science, School of Allied Health, Human Services and Sport, La Trobe University, Melbourne, 3086 Australia; 4 The Wolf Orthopaedic Biomechanics Laboratory, University of Western Ontario, London, Ontario N6A 3K7 Canada; 5 School of Physiotherapy and Exercise Science, Curtin University, Perth, 6102 Australia; 6 School of Health and Rehabilitation Sciences, The University of Queensland, Brisbane, 4072 Australia

**Keywords:** Patellofemoral, Osteoarthritis, Foot orthoses

## Abstract

**Background:**

Foot orthoses have the potential to be an efficacious treatment for patellofemoral osteoarthritis (PFOA) but have not been evaluated in clinical trials in this population. This study aimed to determine the: (i) feasibility of conducting a randomised controlled trial (RCT) investigating the efficacy of foot orthoses in individuals with PFOA; and (ii) effects of foot orthoses versus flat shoe inserts on pain, function, and knee-related quality of life (QOL).

**Methods:**

This 6-week, single-blinded pilot RCT randomly allocated participants with PFOA to receive foot orthoses or flat inserts. The primary outcome of feasibility was determined via the following parameters: one participant recruited per week, 20% (35 h/week) adherence to the intervention, 50% log book completion rate, and < 20% drop-out, with results reported using descriptive statistics. Secondary outcomes included average and maximum pain severity (100 mm visual analogue scale), Anterior Knee Pain Scale, and Knee injury and Osteoarthritis Outcome Score, analysed using analysis of covariance.

**Results:**

Twenty-six participants (16 women; mean (SD) age of 60 (8) years) with PFOA were recruited. All feasibility parameters were exceeded, with three participants recruited per week, > 20% (37.2 [9.8] hours/week) adherence to the intervention, 69.2% (18/26) log-book completion, and 3.8% (1/26) drop-outs. The most common adverse events were arch irritation and shoe fit issues, which were more common in the foot orthoses group (67.9% *versus* 32.1%). There was a trend for the foot orthoses group to report larger improvements in average and maximum pain than the flat insert group, with the mean difference for maximum knee pain severity (21.9 mm, 95% CI − 2.1 to 46.0) exceeding the minimal clinically important difference (15 mm). The estimated sample size for a full-scale RCT is 160 participants. Suggestions to improve study design include a greater number of face-to-face follow-up appointments, a larger variety of foot orthoses to reduce rates of adverse events, and increasing follow-up time to determine long-term efficacy.

**Conclusion:**

This study supports the feasibility of a full-scale RCT to determine the efficacy of foot orthoses *versus* flat inserts in individuals with PFOA.

**Trial registration:**

The trial protocol was retrospectively registered with the Australian and New Zealand Clinical Trials Registry (ANZCTR number: 12616001287426).

## Background

Knee osteoarthritis (OA) is becoming increasingly prevalent [1, 2]. The obesity epidemic, direct joint trauma, and increasing age of the population are often attributed to this increase, with knee OA currently affecting 3.8% of individuals globally [3] and one in 12 Australians (1.9 million individuals) [4]. Pain and stiffness from OA can limit participation in daily activities, regular exercise, and social engagement. More specifically, the burden of patellofemoral osteoarthritis (PFOA) is becoming more evident [5]. The patellofemoral joint (PFJ) is frequently affected by OA before the tibiofemoral joint (TFJ) [6, 7] and can increase an individual’s risk of developing OA in other knee joint compartments [6]. Furthermore, PFOA has a stronger association with symptoms than TFJ OA [8, 9] and tends to occur in younger individuals [10].

Despite the significant burden of PFOA, there is a lack of evidence supporting effective treatments for this condition. Although interventions such as physiotherapy [11, 12] and knee braces [13] have been shown to be effective in the short-term, poor adherence to these treatments limit their long-term effectiveness. Given the large proportion of middle-aged individuals with busy lifestyle commitments (i.e. work and family), interventions used to treat PFOA need to be time efficient, comfortable, and non-invasive to ensure maximum adherence and optimal patient outcomes.

Effective, non-surgical and non-pharmacological interventions are needed to reduce PFOA pain, and the associated impairments in activities of daily living [14]. Prefabricated (off-the-shelf) foot orthoses are relatively inexpensive and accessible for both practitioners and patients, and are an effective treatment for individuals with patellofemoral pain (PFP) [15–18]. Furthermore, there is high adherence [19] and only minor adverse effects associated with the use of foot orthoses in PFP [15]. Given the biomechanical parallels between PFP and PFOA [20, 21], it is possible that interventions used to treat PFP may also be effective for those with PFOA. As such, it is timely to evaluate the efficacy of prefabricated foot orthoses in individuals with PFOA.

The aim of this feasibility study was to explore the key methodological issues for a future large-scale randomised controlled trial (RCT) to estimate the efficacy of foot orthoses in reducing pain and improving function in individuals with PFOA. This was addressed via three key objectives: (i) to determine the immediate comfort levels of foot orthoses versus flat inserts in individuals with PFOA; (ii) to evaluate proof-of-concept for a clinically meaningful benefit in pain and function for foot orthoses compared to flat inserts over 6 weeks; and (iii) to determine whether foot orthoses and flat inserts are credible and acceptable interventions for PFOA.

## Methods

### Study design

This study was a 6-week, participant-blinded, two-arm parallel group randomised controlled feasibility trial. The trial is reported according to the Consolidated Standards of Reporting Trials (CONSORT) 2010 statement: extension for pilot/feasibility studies [22]. The trial protocol was retrospectively registered with the Australian and New Zealand Clinical Trials Registry (ANZCTR number: 12616001287426). Ethical approval was granted by the La Trobe University Human Ethics Committee (S15/286). All participants provided written informed consent prior to enrolment. Ethical standards were in adherence with the National Health and Medical Research Council (NHMRC) National Statement [23].

### Recruitment of study participants

From March 2016 to August 2016, volunteers were recruited from the greater Melbourne community via paid advertisements (e.g. local newspapers, Facebook) and free advertisements (e.g. community newsletters, noticeboards), with a small number of referrals from physiotherapists and podiatrists. Volunteers who responded to advertisements underwent a two-stage screening process by a single investigator (JMT) to determine their suitability for inclusion. Firstly, a preliminary telephone interview or email questionnaire screened for major exclusion criteria. Potentially eligible volunteers were then invited to attend a physical screening appointment at La Trobe University to confirm that eligibility criteria were met. In participants with bilateral PFOA, the most painful eligible knee was selected as the study knee.

### Eligibility criteria

Eligibility for participation in the study was based on the NICE guidelines (https://www.nice.org.uk/guidance/cg177) [24], which stipulate that imaging is not required for a clinical diagnosis of OA. Inclusion criteria were as follows: (i) age 50 to 70 years; (ii) anterior or retropatellar knee pain aggravated by ≥ 2 PFJ-loading activities (stair ambulation, squatting, rising from sitting); (iii) pain during these activities on most days in the past month, and (iv) pain severity ≥ 30 mm on a 100 mm visual analogue scale (VAS) during aggravating activities.

### Exclusion criteria

Volunteers were excluded if they had any of the following: (i) concomitant pain from other knee structures, hip, or lumbar spine; (ii) recent treatment for knee pain (e.g. knee injections or physiotherapy within the previous 3 months; foot orthoses within the previous 12 months); (iii) any foot condition precluding the use of foot orthoses; (iv) knee or hip arthroplasty/osteotomy; (v) neurological or systemic arthritis conditions; (vi) physical inability, or too frail or ill to undertake testing procedures (ascertained via questioning, and clinical examination if needed); or (vii) inability to understand written and spoken English.

### Randomisation

Once eligibility was confirmed, participants were randomly allocated (via concealed allocation) to receive either foot orthoses or flat inserts. The randomisation schedule was generated using an online randomisation program (http://www.randomization.com), in random blocks of 8–12, and the intervention disclosed to the primary investigator (JMT).

### Interventions

**Fig. 1:**
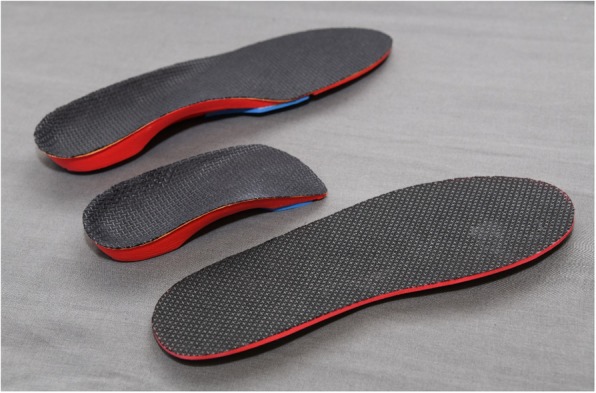
Prefabricated full-length Vasyli foot orthosis (top), prefabricated three-quarter-length Vasyli foot orthosis (middle), and flat insert (bottom)

Interventions were administered by the primary study investigator (JMT), a registered podiatrist with 5 years of musculoskeletal clinical experience. Participants in the foot orthoses group received one pair of commercially available prefabricated full-length foot orthoses (Vasyli Medical, Labrador, Australia) and, if required, one pair of prefabricated three-quarter length foot orthoses that could be accommodated into dress shoes. The foot orthoses were manufactured from ethylene-vinyl acetate (EVA), had inbuilt arch support, and a 6° varus wedge (Fig. 1). We used the high density red (Shore A 75°) EVA product, which is available within the commercial range. The foot orthoses were covered with a synthetic fabric (Cambrelle®, Camtex Fabrics, Cumbria, CA, USA) to ensure no differentiation could be made to the flat insert. If required, devices were moulded to increase comfort, as per our previous RCT [15, 25]. Participants allocated to the flat insert group received a single pair of flat inserts, similar in appearance to the foot orthoses described above (Fig. 1). They were made from the same high density red (Shore A 75°) EVA with identical black Cambrelle® covering fabric. However, the device was uniform in thickness along its full length (3 mm) and had no inbuilt arch support or varus wedging (Fig. 1). It was assumed that the flat insert had some minor cushioning properties, but limited arch support compared to the foot orthoses, and thus could be considered a sham device [26].

### Outcomes and follow-up

Prior to randomisation, patient-reported outcome measures were administered to capture baseline pain and function. Participants were then asked to complete a comfort questionnaire based on the foot orthoses, flat inserts, and their own shoes. Participants were blinded as to which insert was placed within their shoe, and the order of inserts tested was randomised. On completion of the comfort questionnaire, participants were randomised to receive either foot orthoses or flat inserts, and discouraged from using other forms of treatment during the 6-week trial. Participants were also provided with a daily log book to monitor their physical activity, footwear worn, insert wear time, and any adverse events, and were requested to return the log book after week 6. Participants were phoned 7 days after randomisation to ascertain if they were experiencing any difficulties with their allocated inserts, and were invited to return to La Trobe University for any necessary adjustments if required. Follow-up patient-reported outcome measures were conducted at 6 weeks, via paper-based questionnaires mailed to participants with a reply-paid envelope.

#### Anthropometric measures and foot assessments

Anthropometric measures were collected including height, weight, and waist circumference, and body mass index (BMI) was calculated. The Foot Posture Index [27], foot mobility [28], and assessment of the participant’s most frequently worn footwear [29] were also documented at baseline.

#### Primary outcome measure: feasibility of a full-scale RCT

Feasibility was evaluated via recruitment rate, willingness of participants to enrol, number of eligible participants, adherence with allocated shoe insert, log book completion, adverse events (such as blistering, rubbing, and the development of new pain in other body regions), and dropout rate. We set the following parameters to determine feasibility: one participant recruited per week, 20% (35 h/week) adherence to the intervention [30], 50% log book completion rate, and less than 20% dropout rate [25, 31]. The podiatrist who fitted the foot orthoses and flat inserts recorded the number of additional appointments required, prescription notes, and adverse events experienced during fitting and follow-up.

#### Secondary outcome measures: patient-reported outcomes

Patient-reported outcome measures were assessed at baseline and 6 weeks to determine estimates of the effect of foot orthoses compared to flat inserts. These were:

i. Use of rescue medication (e.g. paracetamol) and co-interventions to relieve PFOA pain, documented with a daily-log book throughout the 6-week study period.
ii. Shoe insert comfort [32], measured at baseline in the foot orthoses, flat inserts, and the participant’s own shoes. This was measured using four separate 100 mm VAS: overall shoe insert cushioning, forefoot cushioning, arch cushioning, and heel cushioning. Terminal descriptors were listed as 0 mm = not comfortable and 100 mm = most comfortable imaginable.
iii. Knee injury and Osteoarthritis Outcome Score (KOOS) [33], completed at baseline and 6 weeks. The KOOS is a disease-specific questionnaire with established reliability, validity, and responsiveness in knee OA [34]. The KOOS includes five subscales for pain, symptoms, function in activities of daily living, function in sport/recreation, and knee-related quality of life. A 5-point Likert scale is used to score items from 0 (no problems) to 4 (extreme problems). Scores are transformed to a 0 to 100 scale, with zero representing extreme knee problems and 100 representing no knee problems.
iv. Anterior Knee Pain Scale (AKPS) [35], completed at baseline and 6 weeks. The AKPS consists of 13 items with categories related to limping, weight bearing, walking, stairs, squatting, running, jumping, prolonged sitting with flexed knees, pain, swelling, painful patellar movements, thigh muscle atrophy, and flexion deficiency. Participants select a single response for each item that best describes their knee pain. The 13 individual items are then summated to provide a final score, where 0 represents maximal disability and 100 represents no disability [35]. This scale has established reliability and validity [35–37], and has been recommended for use in studies of PFP [37].
v. Severity of average, worst, and maximum knee pain over the preceding week, measured using a series of 100 mm VAS (terminal descriptors were 0 mm = no pain and 100 mm = worst pain possible). This was measured at baseline and 6 weeks [37]. The VAS were as follows: average pain, worst pain, average pain at rest, average pain during movement, average amount of restriction to your daily activities, maximum pain when walking, maximum pain when sitting for 1 h, maximum pain when rising from sitting, maximum pain when going up and down stairs, maximum pain when squatting, and maximum pain when running. Maximum and average knee pain severity over the previous week was determined using an individual’s most aggravating activity of either ‘rising from sitting’, ‘going up and down stairs’, or ‘squatting’.
vi. Credibility of treatment received was evaluated using the first two items of the Credibility/Expectancy Questionnaire [38].
vii. Global rating of change (GRoC) measured on a 15-point Likert scale (responses ranging from “a very great deal better” to “a very great deal worse”), measured at 6 weeks [39].

### Statistical analysis

Descriptive statistics were used to describe the primary outcome measure of feasibility, which was reported as recruitment rate, number of participants willing to enrol, number of eligible participants randomised, intervention adherence, daily-log book completion, adverse events, dropout rates, and loss to follow-up over the treatment period.

Proof-of-concept analysis: The effect of the intervention on the key secondary outcome (maximum knee pain severity during an individual’s most aggravating activity of either ‘rising from sitting’, ‘going up and down stairs’, or ‘squatting’ in the previous week) was estimated on an analysed as randomised, complete case basis, using linear regression adjusting for baseline values. Estimates of effect were presented as mean differences (MD) in VAS (100 mm scale) with 95% confidence intervals, with proof-of-concept considered as acceptable if the previously reported minimal clinically important difference (MCID) of 15 mm [40] was within the 95% confidence interval (CI). To confirm the appropriateness of parameters utilised in a sample size calculation for the full-scale RCT, the between-person baseline standard deviation (SD) and the within-person correlation between baseline and 6-week measures of the primary outcome were calculated.

The effect of the intervention on the remaining secondary outcomes was estimated on an analysed as randomised, complete case basis, using linear regression adjusting for baseline values (analysis of covariance—ANCOVA). Within-person analyses to establish the immediate comfort levels between the different shoe insert conditions and the participant’s own shoes at baseline were determined using one-way repeated measures analysis of variance and reported as MDs with 95% CIs. Patient-reported GRoC of treatment outcome was reported as median and interquartile range (IQR). All statistical analyses were undertaken using SPSS® version 24.0 (IBM Corp, NY, USA).

Sample size was not formally determined for this feasibility study, as the primary aim was to inform a full-scale RCT with regard to practicalities around recruitment procedures and acceptability of the intervention. However, a sample size of 26 allowed for estimation of key feasibility parameters, such as drop-out of 20%, with reasonable precision (10%), and 80% confidence.

## Results

### Participant demographics and clinical characteristics

Baseline participant characteristics for both treatment groups are presented in Table 1. Those in the flat insert group reported higher levels of usual and worst VAS pain, and a lower AKPS score compared to the foot orthoses group.

**Table 1:** Participant characteristics

	Total group (*N* = 26)	Foot orthoses (*n* = 13)	Flat inserts (*n* = 13)
Age (years)	60 (8)	55 (4)	65 (8)
Number (%) of women	16 (62)	8 (62)	8 (62)
BMI (kg/m^2^)	27.4 (4.7)	25.1 (3.6)	29.7 (4.7)
Right knee affected, *n* (%)	17 (65)	9 (69)	8 (62)
Duration of pain			
3–6 months, *n* (%)	2 (7.7)	–	2 (15.4)
6–12 months, *n* (%)	–	–	–
1–2 years, *n* (%)	2 (7.7)	–	2 (15.4)
≥ 2 years, *n* (%)	22 (84.6)	13 (100)	9 (69.2)
Usual pain VAS (0 to 100 mm)	43 (25)	31 (13)	56 (29)
Worst pain VAS (0 to 100 mm)	57 (30)	49 (26)	66 (31)
AKPS (0 to 100)	51 (18)	61 (12)	41 (18)

Values are reported as mean (SD) unless otherwise stated

*BMI* body mass index, *VAS* visual analogue scale (0 = no pain; 100 = worst pain possible), *AKPS* Anterior Knee Pain Scale (0 = maximal disability; 100 = no disability)

### Recruitment

**Fig. 2:**
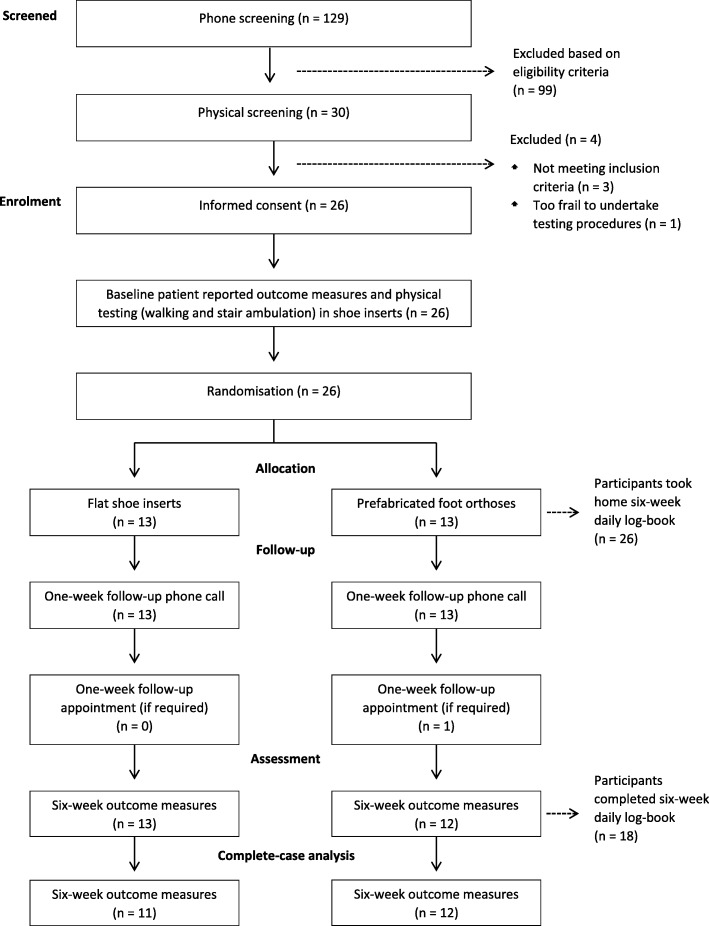
Flow of participants through the study

Participant flow through the study is presented in Fig. 2. Over 5 months, 129 volunteers were screened, of which 30 were eligible. The recruitment rate was three participants per week. Most participants were recruited via print media on community notice boards, in pharmacies, in waiting rooms of doctors’ surgeries and allied health clinics, as well as displays at local markets (Table 2). Four individuals declined to participate due to the time and physical demands of baseline data collection, resulting in a total of 26 being randomised. Thirteen participants were randomised to the foot orthoses group and 13 to the flat insert group.

**Table 2:** Feasibility measures of recruitment

	Total group (*N* = 26)
Recruitment sources, *n* (%)	
Print media (e.g. local newspapers, magazines, posters)	19 (73.1)
Social media (e.g. Facebook, Twitter, Instagram)	3 (11.5)
Referrals from allied health professionals (e.g. physiotherapists, podiatrists)	4 (15.4)

In the foot orthoses group, one participant ceased wearing their allocated intervention due to the development of low back pain, and in the flat insert group, one participant developed gout and ceased wear of their allocated intervention. Both of these participants completed patient-reported outcomes at 6 weeks. One participant in the foot orthoses group was lost to follow-up despite multiple attempts to make contact (3.8% attrition rate), and two participants in the flat insert group had incomplete datasets at 6 weeks. This resulted in 12 participants in the orthoses group and 11 participants in the flat insert group being included in the final 6-week analysis.

### Adherence and completion of daily log book

Adherence to both interventions and completion of the daily log book is reported in Table 3. Overall, participants wore their allocated intervention for a mean (SD) of 37.2 (9.8) hours per week. Participants in the foot orthoses group (37.8 [9.3] hours/week) wore their intervention for slightly longer compared to those in the flat insert group (35.6 [11.6] hours/week). More participants in the foot orthoses group (83.3%) completed their daily log book compared to those in the flat insert group (61.5%).

**Table 3:** Adherence, log book completion rates, adverse events (total single events reported over 6 weeks), use of rescue medication (total single events reported over 6 weeks), and use of co-interventions

	Total group (*N* = 23)	Foot orthoses (*n* = 12)	Flat inserts (*n* = 11)
Adherence (hours/week)*	37.2 (9.8)	37.8 (9.3)	35.6 (11.6)
Completion of daily log book, *n* (%)	18 (72.0)	10 (83.3)	8 (72.2)
Adverse events, *n* (%)	17 (73.9)	11 (64.7)	6 (35.3)
Arch irritation/pain	9 (16.1)	8 (66.7)	1 (9.1)
Back pain	5 (8.9)	3 (25.0)	2 (18.2)
Hip pain	4 (7.1)	3 (25.0)	1 (9.1)
Knee pain	6 (10.7)	4 (33.3)	2 (18.2)
Tightness in footwear/shoe fit issues	9 (16.1)	5 (41.4)	4 (36.4)
General foot discomfort/ache	8 (14.3)	7 (58.3)	1 (9.1)
Rubbing	1 (1.8)	0 (0.0)	1 (9.1)
Too firm	3 (5.4)	2 (16.7)	1 (9.1)
Tired feet	1 (1.8)	0 (0.0)	1 (9.1)
Discomfort/rubbing around MTPJ/toe region	8 (14.3)	6 (50.0)	2 (18.2)
Other (e.g. heel pain, shin pain)	2 (3.6)	0 (0.0)	2 (18.2)
Total	56 (100.0)	38 (67.9)	18 (32.1)
Rescue medication, *n* (%)			
Paracetamol 665 mg	3 (5.4)	0 (0.0)	3 (27.3)
Paracetamol 500 mg	1 (1.8)	1 (8.3)	0 (0.0)
Voltaren (diclofenac sodium)	2 (3.6)	1 (8.3)	1 (9.1)
Celebrex (celecoxib)	2 (3.6)	0 (0.0)	2 (18.2)
Difflam (3% benzydamine hydrochloride topical cream)	1 (1.8)	0 (0.0)	1 (9.1)
Total	9 (100.0)	2 (22.2)	7 (77.8)
Co-interventions, *n* (%)			
Osteopathy	1 (4.0)	0 (0.0)	1 (9.1)
Knee exercises/stretches	2 (8.0)	0 (0.0)	2 (18.2)
Self-massage	1 (4.0)	1 (8.3)	0 (0.0)
Total	4 (100.0)	1 (25.0)	3 (75.0)

*MTPJ* metatarsophalangeal joint

*Values reported as mean (SD)

### Adverse events

There was a total of 56 adverse events reported over the 6-week period, with 17 (73.9%) participants reporting at least one adverse event. The most common adverse events were arch irritation, issues with footwear tightness or shoe fitting, and general foot discomfort/aching. Adverse events were reported more frequently in the foot orthoses group (64.7%) than the flat insert group (35.3%) (Table 3).

### Use of rescue medication and co-interventions

More participants in the flat insert group used rescue medication compared to those in the foot orthoses group (77.8% *versus* 22.2%). There were four reports for the use of co-interventions, with the most common being knee exercises/stretches (Table 3).

### Comfort of interventions

Baseline comfort scores are presented in Table 4. Overall comfort was less whilst wearing the foot orthoses relative to the participants’ own shoe and flat insert. A similar trend was also reported for cushioning in the forefoot, arch, and heel.

**Table 4:** Within-subject comparison of baseline shoe insert comfort recorded with a 100 mm visual analogue scale (VAS)

	Own shoe (*n* = 25)	Foot orthoses (*n* = 26)	Flat inserts (*n* = 24)	Mean difference (95% CI)		
				Foot orthoses vs own shoe	Foot orthoses vs flat inserts	Flat inserts vs own shoe
Overall comfort	73 (26)	60 (28)	75 (21)	− 14 (− 31 to 4)	− 16 (− 33 to 2)	2 (− 15 to 19)
Forefoot cushioning	74 (25)	64 (31)	75 (21)	− 10 (− 28 to 8)	− 12 (− 30 to 6)	2 (− 16 to 19)
Arch cushioning	71 (25)	57 (30)	72 (23)	− 14 (− 32 to 4)	− 16 (− 33 to 2)	2 (− 16 to 19)
Heel cushioning	70 (28)	65 (30)	76 (20)	− 6 (− 24 to 13)	− 11 (− 29 to 7)	6 (− 12 to 23)

Values are reported as mean (SD) unless otherwise stated

100 mm VAS (0 = not comfortable; 100 = most comfortable imaginable)

Note: not all participants were able to complete the comfort questionnaire in all three conditions, due to some participants being unable to complete all three laboratory-based functional tasks (walking and stair ambulation), which was part of a biomechanics study

### Proof of concept

The estimate of effect between groups (mean difference) for the key secondary outcome of maximum knee pain severity during an individual’s most aggravating activity of either ‘rising from sitting’, ‘going up and down stairs’, or ‘squatting’ in the previous week was 21.9 mm (95% CI − 2.1 to 46.0). The SD of this outcome at baseline was 24.6 (19.2 to 35.2), with the correlation between baseline and 6-week measures being 0.72 (0.46 to 0.88). This confirms that a sample size calculation based on an ANCOVA adjusting for baseline value, assuming a between-person SD of 30 mm and baseline to 3-month correlation of 0.5, is appropriate. A sample size calculation using these parameters determined that a sample of 160 (80 per group) would be needed. Allowing for ~ 20% dropouts, this provides a minimum 90% power (*α* = 0.05) to detect a clinically meaningful between-group difference of 15 mm or more in maximum knee pain severity over the preceding week during one of three self-nominated aggravating activities (rising from sitting, stair ambulation, or squatting).

### Clinical outcome measures

At 6 weeks, both groups reported improvements in pain and function (Table 5). The foot orthoses group demonstrated a greater mean change in maximum and average pain severity during the most aggravating activity (of either ‘rising from sitting’, ‘stair ambulation’, or ‘squatting’) in the previous week. Maximum VAS was 21.9 mm (2.1 to 46.0) and average VAS was 15.8 mm (95% CI − 4.9 to 36.6), with the previously reported MCID of 15 mm [40] falling within the 95% CIs. All KOOS subscales were improved in the foot orthoses group compared to the flat insert group.

**Table 5:** Differences in within-group and between-group differences in secondary outcome measures

	Foot orthoses (*n* = 12)			Flat insert (*n* = 11)			
	Baseline	6 weeks	Mean change (6 weeks—baseline)	Baseline	6 weeks	Mean change (6 weeks—baseline)	Foot orthoses—flat inserts adjusted mean difference (95% CI) 6 weeks^^^
Maximum VAS on most aggravating activity (0 to 100 mm)	51.6 (20.3)	24.6 (26.4)	− 27.0 (22.5)	73.2 (25.3)	66.0 (36.9)	− 7.2 (26.0)	− 21.9 (− 46.0 to 2.1)
Average VAS on most aggravating activity (0 to 100 mm)	39.2 (20.0)	14.5 (16.5)	− 24.7 (22.4)	58.8 (25.8)	38.6 (29.1)	− 20.1 (27.7)	− 15.8 (− 36.6 to 4.9)
AKPS (0 to 100)	62.0 (12.2)	72.5 (17.2)	10.5 (14.6)	46.4 (10.9)	49.1 (21.5)	2.7 (17.9)	9.1 (− 8.6 to 26.8)
KOOS—pain (0 to 100)	67.8 (10.8)	79.4 (13.3)	11.7 (13.8)	54.2 (12.5)	60.0 (20.9)	5.8 (15.3)	8.1 (− 6.9 to 23.1)
KOOS—symptoms (0 to 100)	64.6 (18.3)	67.0 (16.6)	2.4 (10.6)	56.1 (15.1)	56.4 (17.8)	0.3 (14.8)	4.4 (− 6.6 to 15.5)
KOOS—ADL (0 to 100)	80.7 (10.6)	87.7 (14.2)	7.0 (8.6)	59.6 (19.8)	56.1 (21.8)	− 3.5 (15.9)	13.7 (0.2 to 27.2)
KOOS—sport/recreation (0 to 100)	37.1 (20.2)	56.3 (30.4)	19.2 (29.0)	15.0 (11.6)	20.0 (21.3)	5.0 (23.9)	25.7 (− 1.7 to 53.0)
KOOS—QOL (0 to 100)	50.5 (12.9)	62.7 (14.4)	12.2 (11.2)	34.2 (16.1)	37.7 (20.0)	3.5 (13.8)	11.3 (− 1.4 to 24.0)

Patient-reported outcomes are reported both within group to describe change over time and between group (foot orthoses versus flat inserts) for differences at 6 weeks

Values are reported as mean (SD) unless otherwise stated

*VAS* visual analogue scale (0 = no pain; 100 = worst pain possible), *AKPS* Anterior Knee Pain Scale (0 = maximal disability; 100 = no disability), *KOOS* Knee Injury and Osteoarthritis Outcome Score (0 = worst score; 100 = best score), *ADL* activities of daily living, *QOL* quality of life

^^^ANCOVA analysis of covariance (adjusted for baseline scores)

### Credibility of treatment

At baseline, both the foot orthoses and flat insert groups had a similar perception that the intervention they had received was credible (item 1: MD − 0.2, 95% CI − 1.4 to 0.9; item 2: − 0.7, − 1.8 to 0.5).

### Global reporting of change

At 6 weeks, participants in the foot orthoses group reported a GRoC median value of 2.5 (min = − 1; max = 6) and those in the flat insert group reported a GRoC median value of 3 (min = 0; max = 6).

## Discussion

This study aimed to investigate whether a full-scale RCT evaluating the effects of prefabricated foot orthoses in individuals with PFOA is feasible. Our secondary aims were to: (i) determine the immediate comfort levels of foot orthoses compared to flat inserts: (ii) evaluate proof-of-concept for a clinically meaningful benefit in pain and function for foot orthoses compared to flat inserts over 6 weeks: and (iii) determine whether foot orthoses and flat inserts are a credible and acceptable intervention for PFOA.

### Primary outcomes

Our findings suggest that a future RCT powered to evaluate the efficacy of foot orthoses *versus* flat inserts in treating PFOA is feasible. Based on observed recruitment rate, adherence, retention, and the calculated sample size to be able to detect clinically meaningful differences in pain and function between the foot orthoses and flat insert groups, a larger-scale RCT is now warranted.

The rate of recruitment for this study was acceptable. It took 5 months to recruit 26 participants at a rate of three participants per week. Recruiting into a larger scale RCT, with a sample size of 160, would be feasible if a longer recruitment period and multiple recruitment sites were employed. This method of recruitment has also been shown to be feasible in one other large-scale RCT investigating the efficacy of foot orthoses in individuals with PFP [41].

After 6 weeks, both groups showed a high level of adherence, with those in the foot orthoses group reporting slightly higher adherence compared to the flat insert group. These high adherence levels are similar to those reported in a previous study of foot orthoses for PFP, where participants reported wearing their foot orthoses for ≥ 60% of the study duration [19]. Furthermore, the high adherence levels may also explain the low dropout rate (3.8%) in this study, which again has been reported in previous foot orthoses for PFP research [15, 16, 18]. In spite of the small loss to follow-up, we have allowed for a 20% dropout rate in our sample size calculation in order to account for the longitudinal study design, which is in line with previously published trial protocol papers investigating shoe inserts in PFP [25] and PFOA [31] populations.

Despite successful recruitment, adherence to the intervention, and study completion, it should be highlighted that 56 adverse events were reported. However, these were minor and transient. Not surprisingly, more participants in the foot orthoses group experienced adverse events compared to the flat insert group, with the most common being arch irritation (16%) and tightness in footwear/shoe fit issues (16%). Arch irritation is common during the initial wear-in period of foot orthoses [15] and has been reported in previous studies of foot orthoses for PFP [15, 16]. However, similar to prior research [15, 16], this did not result in participants ceasing wear of their intervention. In an attempt to maximise adherence and minimise participant dropout, we telephoned all participants 1 week after randomisation to identify and manage any adverse events. Where adverse events could not be resolved over the phone (*n* = 1, 4%), participants were offered a follow up appointment with the primary investigator (JMT). A second phone call was made 1 week later to determine whether further intervention was required. In future full-scale RCTs, we recommend regular follow-up appointments to address any issues as soon as they arise, to potentially minimise adverse events. Furthermore, modifications can be made to the foot orthoses to enhance comfort and reduce the potential for adverse events (e.g. heat moulding, addition of wedges and heel raises), as in our previous RCT [15, 25].

Shoe fit issues were common (16%) in the current study. We attempted to minimise this by providing advice that included altering the type(s) of footwear that participants wore to accommodate the foot orthoses at the baseline appointment. However, there are a number of reasons that participants may not have altered their footwear, including cost [42], occupational requirements [43], and fashion [44]. To combat this issue, in future RCTs, we recommend using a larger selection of prefabricated foot orthoses to allow accommodation into a wider variety of footwear. In addition, we would implement a longer period between providing footwear education and issuing the shoe inserts, to allow participants adequate time to obtain new footwear if required.

### Secondary outcomes

Similar to previous research [45], participants in this study found the foot orthoses to be less comfortable than the flat insert (MD − 16, 95% CI − 33 to 2). This may be due to the arch support of the foot orthoses that may have initially felt uncomfortable for those not familiar with contoured in-shoe devices. Furthermore, the greater comfort of the flat insert may be due to the similarity of these inserts to those that routinely come with footwear. Hence, the flat insert used in the trial may have felt more familiar when compared to the foot orthoses. As such, the flat inserts used in this trial can confidently be used as a comparator in large-scale RCTs investigating foot orthoses as the main intervention of interest.

There was a trend for the foot orthoses group to report larger improvements in their maximum and average VAS pain during their most aggravating activity (of either ‘rising from sitting’, ‘going up and down stairs’, or ‘squatting’ in the previous week) when compared to the flat insert group. These results demonstrated a mean improvement of 21.9 mm (95% CI − 2.1 to 46.0) and 15.8 mm (− 4.9 to 36.6), respectively, which both exceed the previously reported MCID of 15 mm [40]. The improvement that we observed is similar to previous clinical trials evaluating foot orthoses in individuals with PFP [15, 16]. It is plausible that the foot orthoses group experienced greater improvements in pain due to a greater reduction in peak plantar pressures by the arch contouring of the foot orthoses [46], in addition to the slight increase in wear time compared to the flat insert group. Improvements observed over 6 weeks in both treatment groups could be attributed to reductions in plantar pressures [26] and potential shock absorbing properties present within the material of both interventions [26, 47].

At baseline, those in the flat insert group reported higher levels of average and maximum VAS pain during their most aggravating activity over the preceding week, and lower KOOS subscale results, compared to the foot orthoses group. This indicates that those in the flat insert group commenced the trial with a greater amount of knee pain and disability. The randomisation process typically results in an even distribution of baseline characteristics between groups, however, in studies with small sample sizes such as this, despite the randomisation process being rigorously implemented, the discrepancy observed has undoubtedly arisen by chance.

At baseline, there was minimal difference in credibility scores between groups. This may be due to the explanation provided to both groups that they were receiving one of two potential shoe insert interventions to assist with their knee pain. Neither group were informed of the differences between devices, or that one insert may potentially be superior to the other. This demonstrates that the blinding process was effective and that the flat insert used in this trial can be used as an acceptable sham in future RCTs. Both these aforementioned factors are important to consider when planning and conducting large-scale RCTs to assist in reducing resentful demoralisation for those allocated to the control group.

Interestingly, at 6 weeks, both groups reported very similar global improvement scores. Given that the foot orthoses group reported slightly larger improvements in pain and function at 6 weeks, the similarity in GRoC scores may be due to the imbalance in baseline pain and disability scores, in addition to the similarity in perceived credibility of the intervention received at baseline.

### Limitations

There are two key limitations associated with this feasibility study that should be considered. Firstly, due to the small sample size, there was an imbalance in baseline knee pain and disability scores between the two groups, which provides further justification for the need for a larger-scale RCT. Secondly, investigators were not blinded. Although secondary outcome measures were self-reported, thus limiting the ability of researchers to influence participant responses, we recommend that researchers handling data in future trials should be blinded to group allocation, where possible.

Overall, the confidence intervals for mean between-group differences in average and maximum VAS during an individual’s most aggravating activity (of either ‘rising from sitting’, ‘going up and down stairs’, or ‘squatting’ in the previous week) exceeded the known MCID for these outcome measures. This supports our hypothesis that foot orthoses may provide short-term (6-week) clinical benefits to individuals with PFOA. However, it is important that the findings of this feasibility study, with its small sample size, are not interpreted as evidence that definitively supports the use of foot orthoses in this population. Collectively, the feasibility and clinical outcome findings suggest that sufficiently powered RCTs are now required to explore these effects further.

## Conclusion

This study has demonstrated that conducting a larger-scale RCT evaluating foot orthoses for PFOA is feasible in terms of recruitment rates, adherence, participant retention, treatment credibility, and treatment effects. Adequately powered RCTs evaluating the effectiveness of foot orthoses *versus* flat inserts in the treatment of pain, function, and knee-related QOL in individuals with PFOA are now warranted.

## Data Availability

The datasets used and/or analysed for this study are available from the corresponding author on reasonable request.

## Abbreviations

AKPS: Anterior Knee Pain Scale
ANCOVA: Analysis of covariance
CI: Confidence intervals
EVA: Ethylene-vinyl acetate
GRoC: Global rating of change
IQR: Interquartile range
KOOS: Knee injury and Osteoarthritis Outcome Score
MCID: Minimal clinically important difference
MD: Mean difference
NHMRC: National Health and Medical Research Council
OA: Osteoarthritis
PFOA: Patellofemoral osteoarthritis
RCT: Randomised controlled trial
SD: Standard deviation
TFJ: Tibiofemoral joint
VAS: Visual analogue scale(s)
